# Identification of additional /novel QTL associated with resistance to cassava green mite in a biparental mapping population

**DOI:** 10.1371/journal.pone.0231008

**Published:** 2020-04-02

**Authors:** Lydia Ezenwaka, Ismail Rabbi, Joseph Onyeka, Peter Kulakow, Chiedozie Egesi

**Affiliations:** 1 National Root Crops Research Institute, NRCRI, Umudike, Nigeria; 2 International Institute for Tropical Agriculture (IITA), Ibadan, Nigeria; 3 Department of Plant Breeding and Genetics, Cornell University, Ithaca, NY, United States of America; Huazhong University of Science and Technology, CHINA

## Abstract

Cassava green mite [CGM, *Mononychellus tanajoa* (Bondar)] is the most destructive dry-season pest in most cassava production areas. The pest is responsible for cassava fresh root yield losses of over 80%. Deployment of CGM resistant cultivars is the most cost-effective and sustainable approach of alleviating such production losses. The purposes of this study were to validate the stability of CGM resistance genes found in previously published results, to identify new genes for CGM resistance in bi-parental mapping population and estimate the heritability of the trait. A total of 109 F_1_ progeny derived from a cross between CGM resistant parent, TMEB778 and a very susceptible parent, TMEB419 were evaluated under CGM hotspot areas in Nigeria for two cropping seasons. A total of 42,204 SNP markers with MAF ≥ 0.05 were used for single-marker analysis. The most significant QTL (S12_7962234) was identified on the left arm on chromosome 12 which explained high phenotypic variance and harboured significant single nucleotide polymorphism (SNP) markers conferring resistance to CGM and leaf pubescence (LP). Colocalization of the most significant SNP associated with resistance to CGM and LP on chromosome 12 is possibly an indication of a beneficial pleiotropic effect or are physically linked. These significant SNPs markers were intersected with the gene annotations and 33 unique genes were identified within SNPs at 4 – 8MB on chromosome 12. Among these genes, nine novel candidate genes namely; Manes.12077600, Manes.12G086200, Manes.12G061200, Manes.12G083100, Manes.12G082000, Manes.12G094100, Manes.12G075600, Manes.12G091400 and Manes.12G069300 highly expressed direct link to cassava green mite resistance. Pyramiding the new QTL/genes identified on chromosome 12 in this study with previously discovered loci, such on chromosome 8, will facilitate breeding varieties that are highly resistant CGM.

## Introduction

Cassava (*Manihot esculenta* Crantz) is a diploid with a chromosome number of 2n = 36 [1] and highly heterozygous species primarily propagated vegetatively by stem cuttings. A starchy root crop which is widely grown in many parts of sub–Saharan Africa, with Nigeria being the highest producer with an annual production of over 54.8 million tonnes of tuberous roots [2]. More than 50% of Nigeria’s population eats cassava at least once in a day [3]. It serves as a famine reserve crop in the country. Cassava serves as a primary food source and income generation for more than 1 billion people [4,5] including the poorest on the continent. It is also used as animal feed and industrial raw material such as starch and biofuel production [6].

However, the yield of cassava in the African continent has stagnated mostly due to abiotic and biotic stresses. Among the major pests of cassava, cassava green mite (CGM) has been called the most destructive pest of cassava. It has been reported to cause the greatest yield losses in the Americas and Africa [7], especially in the seasonally dry regions of the lowland tropics. High CGM populations caused a reduction of 80% root yield, 50% stem yield and leaf weight, 45% dry matter in the leaves, stems and roots [8].

In recent years, cassava producers spray pesticides to control CGM. The use of pesticides are expensive, harmful to the users and may also disrupt natural enemies like *T*.*aripo* and pest outbreaks [7]. These problems have necessitated an alternative eco-friendly CGM control method. These measures of control against CGM include cultural practices for example early planting to avoid exposing the vulnerable juvenile stages to a significant part of the dry season, biological control using natural predators like *Typhlodromalus aripo* De Leon [9], and host plant resistance [10]. The use of resistant cultivars has been the most successful way of dealing with the destructive effect of CGM in farmers’ fields. This approach not only reduces yield losses but also decreases the levels of pest inoculum in the farming system and thus a more sustainable approach.

Clones with pubescent leaves, stay green ability (SG) and enhanced leaf retention (LR) offer higher levels of resistance to CGM than glabrous clones [11,12]. Earlier studies have detailed the preference of *T*. *aripo*, the natural predator against CGM, for pubescent versus glabrous cassava cultivars infected with *M*. *tanajoa* [13]. It has been studied that leaf trichomes release volatile organic compounds that attract the natural enemies of the herbivores [14], this, therefore, provides shelter for the predatory mite (*T*.*aripo*) and enhances the ability of the predator to find the prey (CGM).

Most studies on CGM have focused on conventional breeding [15,16] but progress has been slow due to the heterozygous nature of the crop, its long growing cycle, lack of suitable donors with resistance, its low seed yield per pollination, and the limited funding for research on this crop [1,17]. The use of molecular markers offers a capable tool for facilitating conventional plant breeding approaches to develop high yielding and pest/disease-resistant cultivars [18]. Identifying molecular markers that are linked to QTL or genes controlling resistance to CGM would facilitate selection for this trait, which has low heritability [11]. Previous QTL mapping studies have reported a few QTL associated with cassava green mite. Two SSR markers, NS1099 and NS346, were identified by Choperna et al. [19] have been associated with CGM resistance. Nzuki et al. [20] also found two QTL for CGM resistance (qCGMc5Ar and qCGMc10Ar) on chromosomes V and X respectively. Furthermore, Ezenwaka et al. [11] identified QTL and candidate genes linked to CGM, LP and LR on chromosome 8 from a genetically diverse population panel. Identification and utilization of new genes for CGM resistance is a major objective of cassava breeding programs to enhance pest resistance and durability.

Biparental populations offer great opportunities for dissecting complex traits in plants and improving crop breeding. The main limitation of using biparental population is that during the population development, it has limited time for recombination events to occur allowing the localization of QTL in large chromosomal regions [21,22]. Information identified by QTL mapping in a biparental population could be useful for incorporating genes into improved cultivars through marker-assisted selection (MAS), map-based cloning of the tagged genes, and for a better understanding of the genetics of complex traits [23]. Commonly used biparental populations for QTL mapping may be produced from the heterozygous F_1_ hybrids, such as F_2_, backcross population, doubled haploid lines, and recombinant inbred line populations [24].

There are four widely used methods for detecting QTL, which are single-marker analysis, interval mapping by maximum likelihood, interval mapping by regression, and composite interval mapping. In this study, the single-marker analysis was used for detecting QTL in a biparental population. The single-marker analysis method is used to detect a QTL near a marker by studying the single-genetic markers one at a time and does not require a complete molecular linkage map [24].

The objectives of this study were to validate the stability of CGM resistance genes found in previously published results, to identify new genes for CGM resistance in bi-parental mapping population and estimate the heritability of the trait. In addition to unravelling the genetics of CGM resistance, this paper also reports on the significance of breeding for CGM resistance for the development of superior genotypes that may greatly improve cassava productivity in Nigeria.

## Materials and methods

### Development of the mapping population

#### Generation of seedlings

The mapping population was developed by crossing two parents with different responses to CGM; TMEB778 is the female parent and TMEB419 is the male parent. TMEB778 is resistant to CGM and high yielding. In contrast, TMEB419 is very susceptible to CGM. These two parent cultivars were chosen for the evaluation because of their commercial relevance in Nigeria. They are also widely deployed in breeding programs to develop new superior genotypes with high yield, pest and disease resistance due to the good end-user qualities of their roots.

Pairwise crossing blocks for TMEB778 and TMEB419 parents were established at the International Institute of Tropical Agriculture (IITA), Ibadan, Nigeria. At flowering (approximately 6 months after planting) the female flowers from TMEB778 were covered with pollination bags early around 8 am to 10 am. The male flowers from TMEB419 with mature pollen were tagged around 11 a.m., collected and hand pollination was done 12 pm to 3 pm in the day. The pollinated flowers were tagged and bagged with pollination bags to protect them from bees carrying foreign pollen; the bags were removed a day later. Seeds matured in 70 to 90 days after pollination. The mature fruits were carefully harvested, placed in labelled pollination bags and were left to shatter naturally.

#### Establishment of the seedling and clonal evaluation trial

Harvested F_1_ botanical seeds were allowed a two—month dormancy period before being sowed in nurseries in February 2014 under screen house conditions at IITA Ibadan. The seeds were sowed in trays filled with sterilized soil with a mixture of loamy and sandy soil in a ratio of 2:1, respectively in the screen house. Seeds germinated quickly at optimal soil temperatures (30 to 35°C) and moisture regimes. They were irrigated twice daily, in the morning and evening. The seeds started germinating from 10 to 12 days after planting and were transplanted when they attained 15 to 20 cm height. After two months in the nursery, F_1_ seedlings were transplanted to a well-prepared field where they were grown and evaluated. Harvesting was done at 12 months after planting in April 2015, after which they were cloned to generate at least 10 stem cuttings per seedling for clonal evaluation.

### Experimental sites

Three locations (Umudike, Igbariam and Otobi) in Nigeria were selected as trial sites for the clonal stage evaluation of the population. Umudike (with annual rainfall of 2200 mm; altitude 120 m; mean annual temperature of 22 to 31°C; coordinates 7^o^24’E, 5^o^29’ N; Dystric Luvisol soils; humid forest); Igbariam (with annual rainfall of 1800 mm; altitude 150 m; mean annual temperature of 24 to 32°C; coordinates 7^o^31’ E, 5^o^56’ N; Dystric Luvisol soils; forest-savanna transition); and Otobi (with annual rainfall of 1500 mm; altitude 319 m; mean annual temperature of 24 to 35°C; coordinates 7^o^20’ E, 8^o^41’ N; Ferric Luvisol soils; southern Guinea savanna) in Nigeria.

### Field layout and experimental design

A total of one hundred and nine F_1_ progeny were cloned in May 2015, were laid out as single-row plots of ten plants with 1m × 1m spacing. The male parent, female parent, and a standard check (IITA TMS 30572) served as the check or control using a randomized incomplete block design. The trial was evaluated in 2015/2016 and 2016/2017 cropping seasons. The clonal trials were harvested at 12 months after planting.

### Fertilizer application

A compound fertilizer (NPK 15:15:15) was applied at the rate of 600 kg ha−^1^. Fertilizer was applied at 8 weeks after planting using the ring method, around the plants to input the fertilizers after planting. The trial was weeded three times during the first 4 months. Weed was controlled traditionally by hand/hoe weeding.

### Agronomic data

The traits evaluated were as follows:

- Cassava Green Mite Severity (CGMS) was evaluated at the visual rating of the damage caused by cassava green mite on a scale of 1 to 5 (S1A Fig). Symptoms rated from 1 = highly resistant; no symptoms observed, 2 = resistant; moderate damage, no reduction in leaf size, scattered chlorotic spots on young leaves, 3 = moderately resistant; severe chlorotic symptoms, slight reduction in leaf size, 4 = susceptible; severe chlorotic symptoms and severe reduction in leaf size of young shoot, 5 = highly susceptible; very severe chlorosis, extensive defoliation, candlestick appearance of young shoots.
- Leaf Pubescence (LP) was characterized visually for the degree of hairiness on the young leaf with 0 = glabrous, 3 = little pubescence, 5 = moderate pubescence and 7 = high pubescence (S1B Fig).
- Stay Green (SG) was scored visually based on a 1–3 scoring scale where: 1 = poor (<50% of the leaves are live and green); 2 = moderately good (50–74% of the leaves are live and green); 3 = very good (≥75% of the leaves are live and green). Leaf longevity was assessed by scoring for SG (S1C Fig).

All the traits were evaluated during the peak of the dry season (January) at six months after planting.

### DNA extraction and SNP genotyping

The DNA extraction was performed using the DNeasy Plant Mini Kit (Qiagen) with slight modifications. The young fresh leaves sample were harvested from the apical part of the cassava plant in the field. About 3–5 tender leaves, weighing about 100 mg– 900 mg, were inserted in the well-labelled extraction tubes arranged in a labelled 96-well box and placed on ice to maintain DNA integrity. From the field, the leaf samples were transferred to the NRCRI molecular laboratory and stored in a -80ºC freezer. Before the commencement of the extraction process, the stored samples were lyophilized for 24 to 48 hours. With the use of a Tissuelyser running with a 1X speed at 1500 strokes/min rate, the samples were ground to a fine powder. Genomic DNA was extracted and quantified using a NanoDrop 1000 (Thermo Scientific) while the molecular weight was assessed with agarose gel electrophoresis.

Genotyping-by-sequencing was performed at the Institute of Genomic Diversity, Cornell University, as described in [25]. The rare-cutting restriction enzyme *Pst*I, which recognizes the sequence CTGCAG, was used. Two barcoded libraries, each consisting of a multiplex of 94 different DNAs from the F_1_ progeny, were each sequenced on one lane of an Illumina HiSeq2000. Parents were sequenced at two times higher coverage compared with the F_1_s to determine allele origin in a large number of SNPs that segregated in the F_1_ progeny. Following sequencing, the reads were filtered for quality and processed using the TASSEL pipeline (www.maizegenetics.net/tassel). This pipeline assigns reads to individuals using the barcode sequences and trims them to 60 bp. Tags (i.e., unique reads) were aligned to version 4.1 of the cassava reference genome (www.phytozome.org/cassava) and SNPs were called by the TASSEL plugin “tbt2Vcf.”

Several filters were used to curate the resulting SNP data before single marker analysis. First, a Chi-square goodness-of-fit test was performed to check for conformance to the expected genotypic frequencies of either a 1:1 ratio (from loci segregating as Aa X aa) or 1:2:1 (from Aa X Aa). Loci that significantly deviated from the expected ratio (*P-*value ≤ 0.05) were removed from further analysis. Also removed were loci with more than 20% missing data across the genotyped individuals as were those with identical recombination information.

### Phenotypic data analysis

The effects of the genotype, location, year, genotype by year, genotype by location, location by year, genotype by location by year interactions were determined for each trait in an analysis of variance (ANOVA) using the standard linear model:

$$Y_{ijkl}=\mu +\beta_{i}+R_{ij}+G_{k}+\beta_{i}\times Gk+e_{ijkl}$$

where *Y*_*ijkl*_ is the phenotypic observations, μ is the mean, β_*i*_ is the effect of the year, *R*_*ij*_ the block effect, *G*_*k*_ the clone effect, β_*i*_ *× G*_*k*_ the interaction between clone by environment, and *e*_*ijkl*_ the residual. Besides, a best-fit linear mixed model for each trait was identified through Bayesian information criterion and used to generate the best linear unbiased predictors (BLUPs) for each individual. Phenotypic mean best linear unbiased prediction (BLUP) was estimated considering the random effects, which represents an estimate of the total genetic value (EGV) for each individual. To calculate the predictor error variance (PEV), the BLUPs were de-regressed using the equation:

$$de-regressed\;BLUP=\frac{BLUP}{1-\frac{PEV}{\sigma_{i}^{2}}}$$

Where PEV is the prediction error variance for each clone and $\sigma_{i}^{2}$ is the clonal variance component [26]. The de-regressed BLUPs were used for the single-marker analysis to help reduce noise variation. The mixed model was computed using the R package *lme4* [27]. The genotypic effects were considered random, while blocks within environments were regarded as fixed effects. Broad-sense heritability (H^2^) for the traits were calculated using the formula $H=\frac{\sigma_{g}^{2}}{\sigma_{g}^{2}+\sigma_{e}^{2}}$ where $\sigma_{g}^{2}$ and $\sigma_{e}^{2}$ are the variance components for the genotype effect and the residual error, respectively, on a plot basis.

The phenotypic correlations were calculated between traits using trait means of the clones and this was performed using Pearson’s correlation coefficient.

### Mapping of QTL

A total of 42,204 SNP markers with MAF ≥ 0.05 were used for single-marker analysis. To identify significant QTL, the single marker analysis was performed using the generalized linear model (GLM) implemented in the TASSEL 5.0 [28]. Genome-wide significance (α = 5%) for declaring a QTL was determined using a permutation test (1000 replications) for each trait.

GLM was used to calculate *P*-values for associating each marker with the traits evaluated using TASSEL 5.0 [28].

The statistical formula for the GLM is *y* = *Xb* + *e* [29]

Where y is the vector of the phenotypic observations, *X* is the known design matrix, *b* is a vector containing the fixed effects (genetic marker information), and *e* is the vector of random residues. Manhattan plots were generated using the R package qqman [30].

### Candidate genes identification approach

The QTL peaks were scanned for potential candidate genes underlying the studied traits based on the gene ontologies and predicted functions. The gene ontology annotation was done using Panther (http:/go. Pantherdb.org/). These sequences were aligned against the cassava V6 reference genome assembly using the intersect function from bedtools [31].

## Results

### Phenotypic evaluation of the mapping population

Descriptive statistics of phenotypic data obtained for two growing seasons 2015/2016 and 2016/2017 in the three locations; Igbariam, Otobi and Umudike are presented in Table 1. The mean of CGM severity was 2.12 (on a scale of 1–5) across all the genotypes evaluated in the three locations. Otobi had the highest mean CGM severity (2.24) while Umudike has the lowest mean CGM severity of 1.80. The highest mean for leaf pubescence (LP) and stay green (SG) were recorded in Igbariam while the lowest mean was found in Otobi. This implies that the more the leaf pubescent and stay green ability the less the severity of CGM.

**Table 1:** Descriptive statistics (mean ± standard error of the mean (SEM), coefficient of variation (CV) and broad-sense heritability estimates (H^2^) for phenotypic data across locations and years.

	Igbariam			Otobi			Umudike			Pooled		
Traits	Mean ± SEM	CV	H^2^	Mean ± SEM	CV	H^2^	Mean ± SEM	CV	H^2^	Mean ± SEM	CV	H^2^
**CGMS**	2.31 ± 0.09	41.44	0.45	2.24 ± 0.06	30.30	0.32	1.80 ± 0.07	41.57	0.42	2.12 ± 0.04	39.28	0.32
**LP**	4.43 ± 0.25	62.39	0.21	3.78 ± 0.09	27.30	0.17	4.23 ± 0.24	62.56	0.23	3.93 ± 0.25	73.23	0.24
**SG**	2.49 ± 0.05	20.84	0.58	2.18 ± 0.05	22.01	0.27	2.48 ± 0.06	34.11	0.42	2.48 ± 0.03	22.30	0.32

CGMS, cassava green mite severity; LP, leaf pubescence; SG, stay green

Variabilities for the traits evaluated for the two growing seasons in the three locations were estimated by the coefficient of variation (CV), ranged from 22.30% for SG to 73.23% for LP. Broad-sense heritability estimates (H^2^) ranged from 0.24 to 0.32. Heritability estimates were highest for CGMS and SG (0.32) and the least heritable trait was LP (0.24).

#### Distribution and correlation analysis between traits in the mapping population

**Fig 1:**
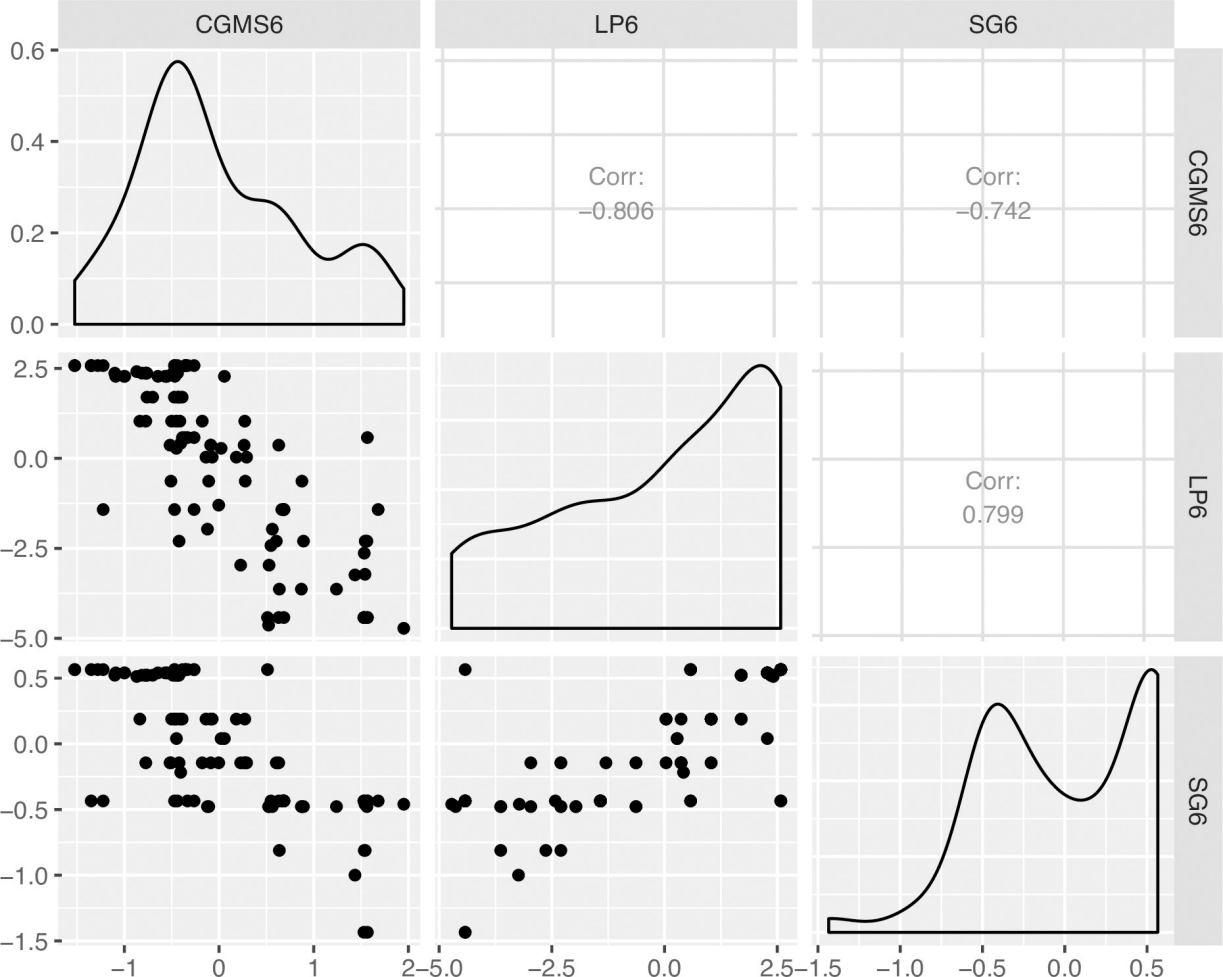
Phenotypic distribution and correlation coefficients for cassava green mite severity and other associated traits.

The phenotypic correlation among the different traits evaluated in three locations in two years is presented in (Fig 1). Results showed that the LP (P ≤ 0.001, r = -0.81) and SG (P ≤ 0.001, r = -0.74) were significantly and negatively correlated with CGMS. A significantly positive result between LP and SG (r = 0.80). The results also showed that plants with severe CGM had glabrous to little leaf pubescent and poor stay green.

Generally, most of the genotypes tend to be resistant to CGM, high leaf pubescence and outstanding stay green ability (Fig 1).

#### Analysis of variance for phenotypic traits

The combined ANOVA of all traits for the three locations across two years showed a significant difference (P < 0.001, P < 0.01 and P < 0.05) for the location effect (Table 2). This indicates the presence of substantial environmental variance. Genotype by location interaction was significant (P < 0.05) for CGMS and LP. Genotype effect was only significant (P < 0.05) for CGMS and LP. Genotype by location by year interactions were not significant for all the traits. Apart from SG, all other traits were significant for CGMS and LP for genotype by year interactions.

**Table 2:** Analysis of variance (ANOVA) for each trait across the three locations and two years.

Traits	Source of variation	Df	MS	Pr(>F)
**CGMS**	Genotype	108	1.08	**
	Location	2	24.12	***
	Year	1	21.07	***
	Genotype: Location	212	0.46	*
	Location: Year	2	12.64	***
	Genotype: Year	105	0.37	*
	Genotype: Location: Year	123	0.42	ns
**LP**	Genotype	108	9.79	**
	Location	2	813.95	***
	Year	1	16.24	**
	Genotype: Location	212	6.64	*
	Location: Year	2	20.78	**
	Genotype: Year	105	3.11	*
	Genotype: Location: Year	123	3.51	Ns
**SG**	Genotype	108	0.48	ns
	Location	2	3.90	***
	Year	1	0.09	ns
	Genotype: Location	212	0.41	ns
	Location: Year	2	0.18	ns
	Genotype: Year	105	0.20	ns
	Genotype: Location: Year	123	0.18	ns

DF, degrees of freedom; MS, Mean square; F‐probabilities are indicated by symbols:

*P<0.05

**P<0.01

***P<0.001, ns (non-significant). CGMS, Cassava Green Mite Severity; LP, Leaf Pubescence; SG, Stay Green.

### Single marker analysis

For the combined dataset, 293 significant SNP markers were identified for CGM, LP and SG. Single marker analysis identified 95, 71, and 127 significant markers for cassava green mite resistance, leaf pubescence and stay green respectively. The most significant SNP marker (S12_7962234) has a–log_10_ (p-value) of 8.91 and explained 31% of observed phenotypic variation. The contribution of any single SNP marker to the phenotypic variation ranged from 18 to 31% across the traits (S1 Table).

#### Cassava green mite severity

**Fig 2:**
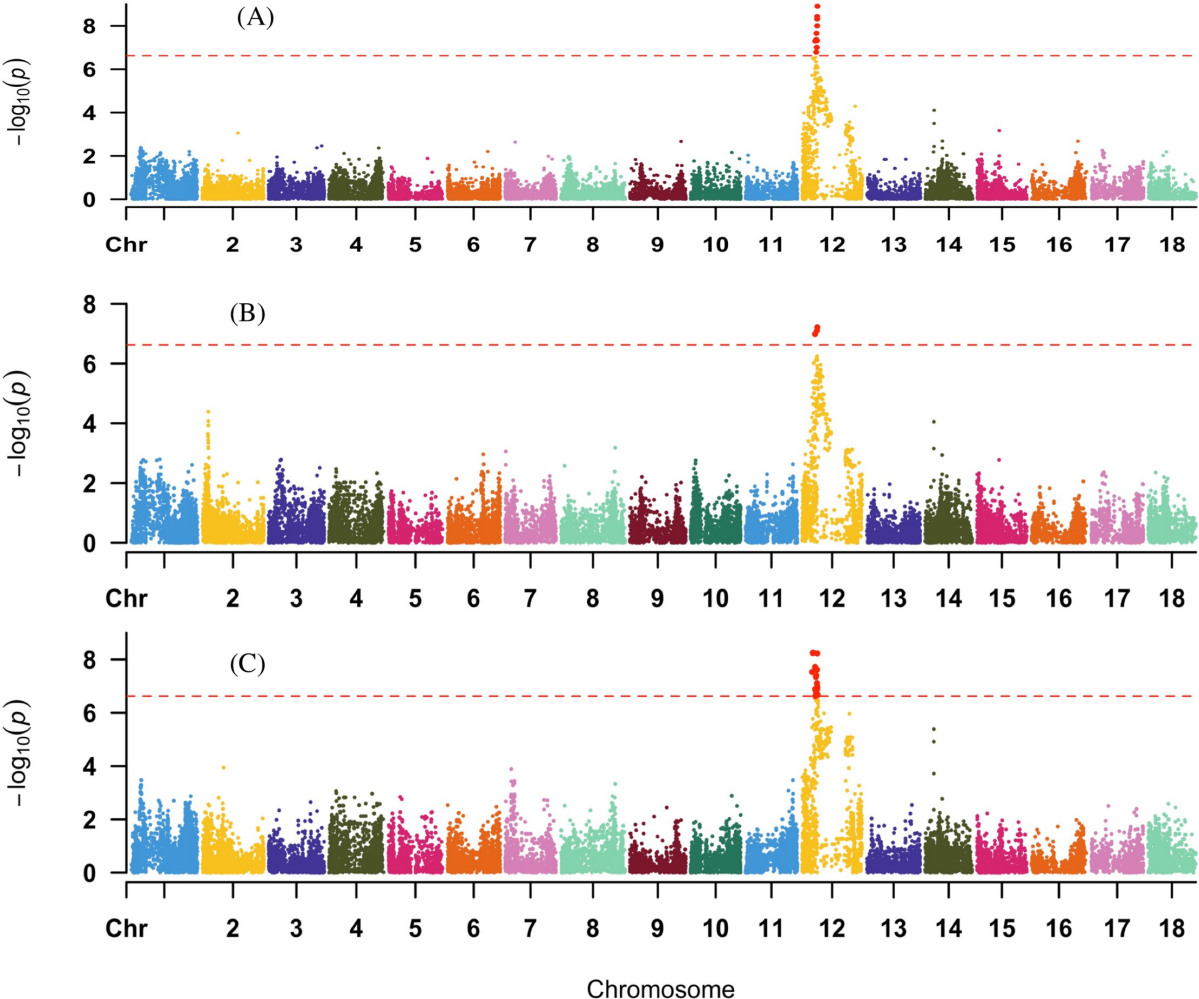
QTL associated with (A) cassava green mite resistance, (B) leaf pubescence and (C) Stay green on chromosome 12 across the three locations in two years.

Ninety-five markers were significantly associated with CGM resistance (S1 Table). The significant markers associated with the trait mostly concentrated at a single region of the left arm side on chromosome 12 (Fig 2A). The top significant SNP marker (S12_7962234) explained 31% of the phenotypic variance (S1 Table). Variance explained by the significant markers ranged from 18 to 31%.

#### Leaf pubescence

Seventy-one markers were found to be significantly associated with leaf pubescence (S1 Table). The significant markers associated with this trait also lies on chromosome 12 (Fig 2B). The top significant SNP marker (S12_7962234) explained 26% of the phenotypic variance (S1 Table). Variance explained by the significant markers ranged from 18 to 26%.

#### Stay green

The study showed that one hundred and twenty-six markers were found to be significantly associated with stay green (S1 Table). Chromosome 12 harbours the top significant SNP marker (S12_5524524) associated with the trait (Fig 2C). This marker explained 30% of the phenotypic variation (S1 Table). Variance explained by the significant markers ranged from 20 to 30%.

However, the same SNPs on chromosome 12 were significantly associated with more than one trait when mapping across all clones in the population. This could be as a result of pleiotropy or closely linked genes. Here, in this study, chromosome 12 had associations for CGMS, LP and SG.

### Candidate genes

The significant SNPs markers were intersected with the gene annotations and 33 unique genes were identified within SNPs associated with CGMS, LP and SG at 4 – 8MB on chromosome 12 (S2 Table). Among these genes, nine candidate genes highly expressed direct link to cassava green mite resistance. In Table 3, the nine candidate genes were sectioned into seven groups according to the protein structure: trichome birefringence related protein, ethylene-responsive, MYB, disease resistance protein family, homeodomain, zinc finger and pentatricopeptide transcription factor genes. These nine candidate genes are classes of membrane proteins that function in multicellular trichome development, plant growth, inflammatory and immune responses in organisms.

**Table 3:** Candidate genes associated with CGM resistance.

Gene	Chr	Gene description	Gene function
**Zinc finger transcription factor genes**			Initiation of inflorescence trichomes
Manes.12G077600	12	Zinc-finger 8-type family protein	
Manes.12G086200	12	C2H2-type-zinc-finger-family-protein	
**Pentatricopeptide repeat family**			Function in plant defense
Manes.12G061200	12	Pentatricopeptide-repeat-(PPR)-superfamily-protein	
**MYB-domain transcription factor genes**			Trichome differentiation
Manes.12G083100	12	MYB-domain-protein-4	
Manes.12G082000	12	MYB-like-102	
**Disease-resistance-protein-family**			Disease-resistance
Manes.12G094100	12	Disease-resistance-protein-(CC-NBS-LRR-class)-family	
**Homeodomain transcription factor genes**			Trichome differentiation and leaf proximodistal axis
Manes.12G075600	12	Homeodomain-glabrous-2	
**Trichome Birefringence related protein**			Trichomes and stem development
Manes.12G091400	12	Protein trichome birefringence-related	
**Ethylene-responsive transcription factors**			Increase the number of cells per trichomes
Manes.12G069300	12	Ethylene-forming-enzyme	

Chr, chromosome.

## Discussion

The study demonstrated the identification of new QTL in a biparental population with reasonably strong effects on resistance to cassava green mite, leaf pubescence and ability to stay green.

Host plant resistance has been an effective method of controlling CGM. It aims at developing varieties with certain plant attributes like stay green ability, leaf pubescence and leaf retention that enables cassava plant to suppress the initial build-up of CGM population, prevent CGM attack and favours the colonization of biological control agents such as *T*. *aripo*. The result of this study showed that leaf pubescence and stay green significantly were negatively correlated with cassava green mite severity. This means that genotypes with excessive pubescent leaves and outstanding stay green ability tend to be resistant/tolerant to cassava green mite attack. The association of leaf and shoot pubescence, stay green ability, leaf retention and cassava green mite have been reported by Ezenwaka et al. [11]. Pubescence offers an excellent level of resistance to *Mononychellus* mites and also helps biological control agents (*T*. *aripo*) to colonize the plant [12].

The 109 genotypes evaluated showed good resistance to the pest although with a differential response at the peak of the pest pressure. Three types of pest response were observed in the genotypes evaluated, namely, (i) highly resistant genotypes, (ii) resistant genotypes and (iii) moderately resistant genotypes. Both the highly resistant and resistant genotypes are generally low in pest symptoms with the former almost symptomless during the peak of the pest pressure. The moderately resistant genotypes had severity symptoms rated above 2 at peak of pest pressure but recovered after the dry season with the genotypes showing fewer pest symptoms. The pest response profile of the moderately resistant genotypes is an indication of a good and efficient gene in the pest response pattern.

Determining broad-sense heritability estimates increases our basic understanding of the amount of genetic variance expressed as a proportion of the total phenotypic variance. The broad-sense heritability estimates for the traits evaluated ranged from 0.24 to 0.32, for traits would be mild to moderately heritable as reported by [11]. This indicates that a large amount of the observable variance in the traits is due to non-genetic effects. Marker-assisted selection could help to improve the selection response of these low heritability traits for cassava improvement.

### Comparison of the novel QTL/genes with previously mapped QTL

Single marker analysis is a good choice when the goal is for the detection of a QTL linked to a marker [32]. The present study using this approach identified 293 significant SNP markers on chromosome 12, which are linked to CGM resistance, leaf pubescence and stay green.

The QTL position identified in this study was compared with those identified in previous studies that have been associated with resistance to cassava green mite. Choperna et al. [19] have mapped two SSR markers (NS1009 and NS346), with strong phenotypic effect (R^2^ of 32% to 37%) in a backcross population. Additional two QTL (qCGMc5Ar and qCGMc10Ar) on chromosome 5 and 10 were identified in biparental population with LOD of 20.19 and R^2^ of 6.48% and a LOD of 20.43 and R^2^ of 4.11% respectively by Nzuki et al. [20]. Ezenwaka et al. [11], identified the most significant SNP marker S8_5962253, R^2^ of 6.9% on chromosome 8 QTL region in a diverse population, they also found 17 candidate genes (Manes.08G058500, Manes.08G048200, Manes.08G048800, Manes.08G034200, Manes.08G046400, Manes.08G041900, Manes.08G026500, Manes.08G053900, Manes.08G060500, Manes.08G058000, Manes.08G045400, Manes.08G035100, Manes.08G043900, Manes.08G024700, Manes.08G046700, Manes.08G044000, Manes.08G026900) that have a strong association with genes conferring resistance to insects/pests in plants. The most significant marker S12_7962234, R^2^ of 31% on chromosome 12 identified for CGM resistance genes (Manes.12077600, Manes.12G086200, Manes.12G061200, Manes.12G083100, Manes.12G082000, Manes.12G094100, Manes.12G075600, Manes.12G091400 and Manes.12G069300) in this study was at a different genomic location as compared to those identified in previous studies. None of those QTL has the same map location with the QTL identified here, suggesting that these genes/QTL are novel and should be targeted for pyramiding via marker-assisted breeding. Since the most significant QTL identified has a high phenotypic variance, this locus could be useful for QTL/gene pyramiding. In this study, the QTL associated with CGM resistance and LP corresponding to the most significant SNP marker (S12_7962234) were co-localized on chromosome 12 and also shared a common genomic region on the left arm of chromosome 12. These could be as a result of beneficial pleiotropic effects. This finding also supported the strong correlations observed among these traits. A recent publication by Ezenwaka et al. [11] reported favourable pleiotropic effects as genes conferring resistance to CGM were linked to leaf pubescence and leaf retention. However, these desirable traits (leaf pubescence and stay-green) may be introduced along with pest resistance into susceptible and glabrous varieties. These QTL are now the focus for more detailed analysis to get better insights into mechanisms of resistance to CGM, and also for breeding purposes. This functional understanding may also be important to identify and validate candidate genes underlying these QTL.

In this study, a total of nine genes (Manes.12077600, Manes.12G086200, Manes.12G061200, Manes.12G083100, Manes.12G082000, Manes.12G094100, Manes.12G075600, Manes.12G091400 and Manes.12G069300) were identified as candidates encoding crucial transcription factors associated with multicellular trichome development, inflammatory and immune responses within 4 to 8 Mb region of the significant markers on chromosome 12. The candidate genes identified in this study have been associated with trichome development in the previous study of Ezenwaka et al. [11] expect for the disease resistance protein gene family found in this study. These novel candidate genes provide useful information to study the relevant molecular networks of multicellular trichome development and pest/disease responses in cassava. The transcription factors are zinc finger, pentatricopeptide, MYB, disease resistance protein family, homeodomain, trichome birefringence related protein and ethylene-responsive. Zinc finger transcription factor genes are associated with phytohormones to regulate multicellular trichome development in *Cucumis sativus* (Cucumber) [33]. In plant kingdom, responses to environmental conditions and developmental regulation of floral meristems, vascular systems and lateral organs including trichome development all involve homeodomain-leucine zipper transcription factor genes [33]. Trichome birefringence-related protein contribute to the synthesis and deposition of secondary wall cellulose in trichomes and stem development [34]. This gene may also be involved in insects/herbivores control, leaf temperature and transpiration maintenance [33]. MYB-domain transcription factor genes, a MYB-like HTH regulates floral papillae, leaf proximodistal axis development and trichome differentiation in *Nicotiana tabacum* (tobacco) [35]. Ethylene–responsive transcription factor genes are involved in the stimulation of epidermis cell division in *Cucumis sativus*, resulting in aberrant guard cell and trichome formation [36]. The pentatricopeptide repeat (PPR) family has been found to function in plant defence [20]. Given the importance of cassava in Africa, breeding for disease resistance is very essential, as the most common disease resistance genes belonging to the nucleotide-binding sites (NBS) and the leucine-rich repeat (LRR) (NBS-LRR) was found as one of the candidate genes. This gene family functions in inflammatory and immune responses of organisms [37]. No functional resistance genes have been cloned in cassava [37]; nevertheless, genes found in this study have strong homology with earlier reported genes from other species.

## Conclusion

Identification of different pest resistance gene is critical in providing stable and durable resistance and efforts in this direction to identify additional QTL to CGM resistance are expected to continue in the African germplasm. Our results indicated a new QTL region on chromosome 12 which led to the identification of nine candidate genes that appear to be associated with cassava green mite resistance, leaf pubescence and stay green. These QTL/genes are suitable targets for successive fine mapping and gene cloning to develop gene-based SNP markers. Pyramiding the novel QTL identified in this study with earlier discovered loci, such as chromosome 8, will result in the rapid development of superior cultivars with CGM resistance and good productivity.

## Supporting information

S1 Fig Pictures of the three traits evaluated. (A) CGMS symptoms rated on a scale 1 to 5. (B) LP was characterized on a scale of 0, 3, 5 and 7 (C) SG was scored based on a 1–3 scoring scale.
(TIF)

S1 Table Summary of single marker analysis with significant markers associated with CGM resistance, LP and SG. (XLSX)

S2 Table Details of 33 genes annotated as candidates associated with CGM resistance, leaf pubescence and stay green ability. (XLSX)
