# Supplementary figures and images for: Identification of additional /novel QTL associated with resistance to cassava green mite in a biparental mapping population
Source: PLoS One. 2020 Apr 2;15(4):e0231008. doi: 10.1371/journal.pone.0231008 (PMC7117712; doi:10.1371/journal.pone.0231008)

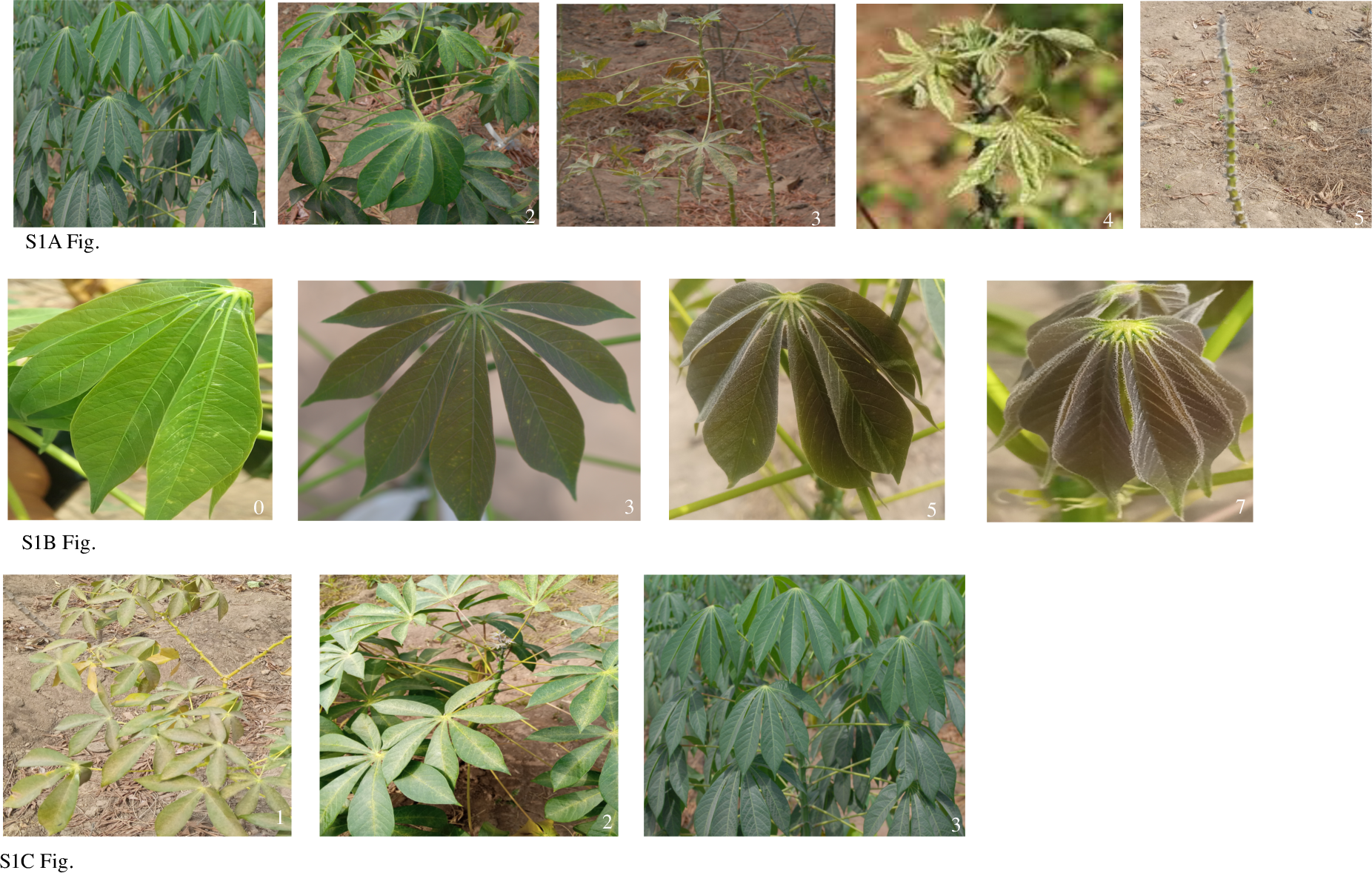

Supplement: S1 Fig — (A) CGMS symptoms rated on a scale 1 to 5. (B) LP was characterized on a scale of 0, 3, 5 and 7 (C) SG was scored based on a 1–3 scoring scale. (TIF) [file pone.0231008.s001.tif]
